# Protein destroyer: Inducible E3-DART for targeted protein degradation in plants

**DOI:** 10.1093/plcell/koae114

**Published:** 2024-04-10

**Authors:** Nitin Uttam Kamble

**Affiliations:** Assistant Features Editor, The Plant Cell, American Society of Plant Biologists; John Innes Centre, Norwich Research Park, NR4 7UH, UK

The CRISPR-Cas9 genome editing system has revolutionized biology. It is most widely used to functionally deactivate genes by targeted mutation, something that would be classified as a pre-translational strategy. However, a powerful alternative to attenuate or eliminate gene functions at the post-translational stage is the targeted degradation of a protein of interest.

Animal and plant cells have evolved dynamic protein quality control mechanisms during development and in response to environmental signals. An example of such a mechanism is the ubiquitin-proteasome system (UPS) that involves an ATP-dependent enzymatic cascade consisting of an E1 ubiquitin-activating enzyme, an E2 ubiquitin-conjugating enzyme, and an E3 ubiquitin ligase that plays a crucial role in providing substrate specificity for ubiquitylation (Scheffner et al. 1995).

Synthetic degrons, engineered sequences within a protein that mark it for degradation by the UPS, have been widely exploited in cell biology but not in plants. For instance, the introduction of ligand-based degrons induced by the phytohormones such as auxin and coronatine are extensively used in yeast and animals (Nishimura et al. 2009). These engineered degrons can be specifically induced in organisms that do not naturally produce phytohormones, but not in plants. Another system, called proteolysis-targeting chimera, cannot be used for proteins for which a specific ligand is not known since it needs a small bifunctional probe that binds specifically to the POI and an E3 ligase (Sakamoto et al. 2001). Temperature-sensitive degrons can conditionally inactivate the targeted protein at 37 °C, but they cannot be broadly used in plants due to this high temperature requirement (Dohmen et al. 1994).

To address this gap, **Linzhou Huang and Marcela Rojas-Pierce** (Huang and Rojas-Pierce. 2024) developed an inducible degron system for plant protein depletion. Their system uses the well-characterized interaction between the Salmonella secreted protein H1 (SspH1) and the human protein kinase N1 (PKN1) (Keszei et al. 2014). SspH1 has E3 activity (via its Novel E3 Ligase [NEL]-domain) and is able to bind Homology Region 1b (HR1b) domain of PKN1 (via its LRR domain). The interaction between SspH1 and HR1b results in the ubiquitylation of PKN1, thereby targeting the PKN1 for degradation (Figure). By leveraging this system, Huang and Rojas-Pierce (2024) have developed a new tool called E3-targeted Degradation of Plant Proteins (E3-DART) to degrade target proteins in planta. The E3-DART protein consists of the NEL and LRR domains of SspH1, as well as an mCherry fluorescent protein for visualization. The target protein (for the proof-of-principle, the authors used GFP) is tagged with the HR1b domain of PKN1 (HR1b-GFP) (Figure). Transient and stable coexpression of E3-DART and HR1b-GFP in *Nicotiana benthamiana* or *Arabidopsis thaliana* resulted in minimal accumulation of HR1b-GFP, indicating that the HR1b-GFP fusion was indeed targeted for degradation by the synthetic E3-DART. An inactive version of the enzyme, E3-DART^C492A^, or a target protein that could no longer be bound by E3-DART (HR1b^R181/185A^-GFP) abolished this targeted degradation, further supporting that E3-DART is indeed working as intended.

**Figure.**
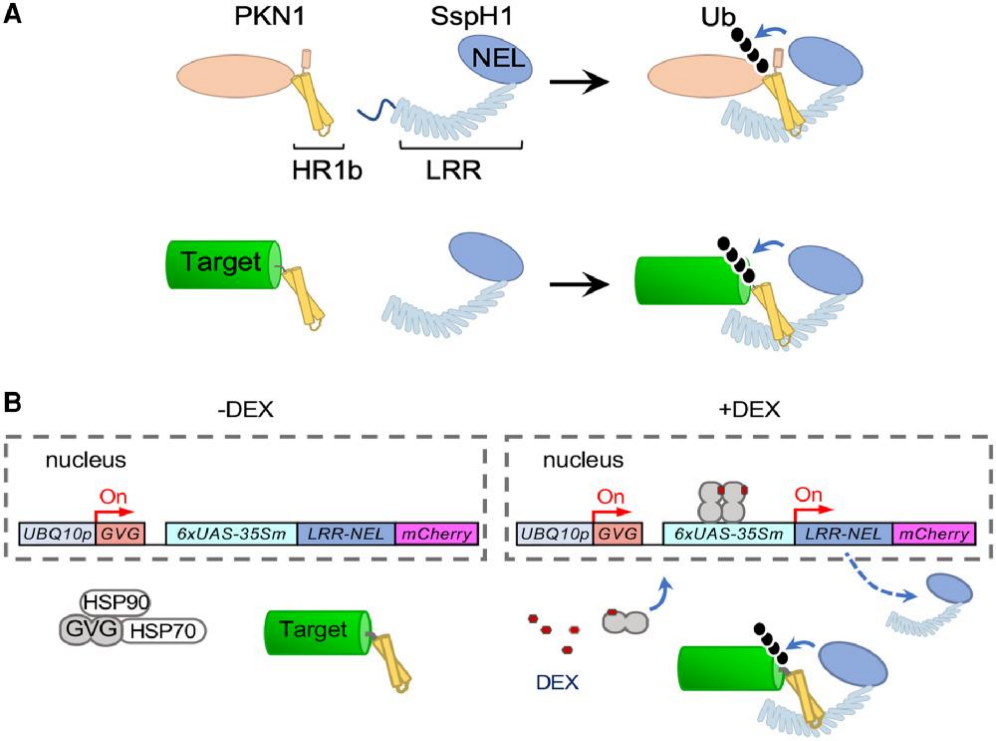
Inducible degron system for plant proteins. **A)** E3-DART system consisting of interaction domain (LRR) that interacts with HR1b from PKN1 fused to a target protein for ubiquitylation by E3 ligase activity of NEL. **B)** Dexamethasone (DEX)-inducible system controlling the expression of the NEL E3 ligase chimera with the GVG transactivation system. Reprinted from Huang and Rojas-Pierce. (2024) Figure 1A and 4A.

The authors confirmed that degradation of the targeted protein occurs via the ubiquitin-proteasome pathway by blocking the process via the 26S-proteasome inhibitor MG132. Moreover, Huang and Rojas-Pierce (2024), detected high molecular-weight ubiquitin conjugates in HR1b-GFP upon MG132 treatment. They demonstrated that E3-DART works efficiently with both nuclear and cytosolic proteins from Arabidopsis.

To enhance the robustness and functionality of E3-DART, the authors developed an inducible system using the GAL4-VP16-GR (GVG) transcription induction system (Figure) and observed the degradation of HR1b-GFP as early as 3 h after dexamethasone in transient assays. Such an inducible technology was lacking in plants, and this robust system should facilitate many studies, as inducible protein degradation systems have advanced research in yeast and animal cells.
